# Activation of Liver FGF21 in hepatocarcinogenesis and during hepatic stress

**DOI:** 10.1186/1471-230X-13-67

**Published:** 2013-04-17

**Authors:** Chaofeng Yang, Weiqin Lu, Tao Lin, Pan You, Min Ye, Yanqing Huang, Xianhan Jiang, Cong Wang, Fen Wang, Mong-Hong Lee, Sai-Ching J Yeung, Randy L Johnson, Chongjuan Wei, Robert Y Tsai, Marsha L Frazier, Wallace L McKeehan, Yongde Luo

**Affiliations:** 1 Center for Cancer and Stem Cell Biology, Institute of Biosciences and Technology, Texas A&M Health Science Center, 2121 W. Holcombe Blvd., Houston, TX, 77030-3303, USA; 2 IBT Proteomics and Nanotechnology Laboratory, Institute of Biosciences and Technology, Texas A&M Health Science Center, 2121 W. Holcombe Blvd., Houston, TX, 77030-3303, USA; 3 Department of Molecular Pathology, The University of Texas M.D. Anderson Cancer Center, Houston, TX, 77030, USA; 4 Department of Molecular and Cellular Oncology, The University of Texas M.D. Anderson Cancer Center, Houston, TX, 77030, USA; 5 Department of Emergency Medicine, The University of Texas M.D. Anderson Cancer Center, Houston, TX, 77030, USA; 6 Department of Endocrine Neoplasia and Hormonal Disorders, The University of Texas M.D. Anderson Cancer Center, Houston, TX, 77030, USA; 7 Department of Biochemistry and Molecular Biology, The University of Texas M.D. Anderson Cancer Center, Houston, TX, 77030, USA; 8 Department of Epidemiology, The University of Texas M.D. Anderson Cancer Center, Houston, TX, 77030, USA

**Keywords:** Adipose tissue, Biomarker, Endocrine FGF, Fibroblast growth factor 21 (FGF21), Hepatic expression, Hepatocellular carcinoma, Hepatocytes, Liver disease, Metabolism

## Abstract

**Background:**

FGF21 is a promising intervention therapy for metabolic diseases as fatty liver, obesity and diabetes. Recent results suggest that FGF21 is highly expressed in hepatocytes under metabolic stress caused by starvation, hepatosteatosis, obesity and diabetes. Hepatic FGF21 elicits metabolic benefits by targeting adipocytes of the peripheral adipose tissue through the transmembrane FGFR1-KLB complex. Ablation of adipose FGFR1 resulted in increased hepatosteatosis under starvation conditions and abrogation of the anti-obesogenic action of FGF21. These results indicate that FGF21 may be a stress responsive hepatokine that targets adipocytes and adipose tissue for alleviating the damaging effects of stress on the liver. However, it is unclear whether hepatic induction of FGF21 is limited to only metabolic stress, or to a more general hepatic stress resulting from liver pathogenesis and injury.

**Methods:**

In this survey-based study, we examine the nature of hepatic FGF21 activation in liver tissues and tissue sections from several mouse liver disease models and human patients, by quantitative PCR, immunohistochemistry, protein chemistry, and reporter and CHIP assays. The liver diseases include genetic and chemical-induced HCC, liver injury and regeneration, cirrhosis, and other types of liver diseases.

**Results:**

We found that mouse FGF21 is induced in response to chemical (DEN treatment) and genetic-induced hepatocarcinogenesis (disruptions in LKB1, p53, MST1/2, SAV1 and PTEN). It is also induced in response to loss of liver mass due to partial hepatectomy followed by regeneration. The induction of FGF21 expression is potentially under the control of stress responsive transcription factors p53 and STAT3. Serum FGF21 levels correlate with FGF21 expression in hepatocytes. In patients with hepatitis, fatty degeneration, cirrhosis and liver tumors, FGF21 levels in hepatocytes or phenotypically normal hepatocytes are invariably elevated compared to normal health subjects.

**Conclusion:**

FGF21 is an inducible hepatokine and could be a biomarker for normal hepatocyte function. Activation of its expression is a response of functional hepatocytes to a broad spectrum of pathological changes that impose both cellular and metabolic stress on the liver. Taken together with our recent data, we suggest that hepatic FGF21 is a general stress responsive factor that targets adipose tissue for normalizing local and systemic metabolic parameters while alleviating the overload and damaging effects imposed by the pathogenic stress on the liver. This study therefore provides a rationale for clinical biomarker studies in humans.

## Background

Fibroblast growth factor 21 (FGF21) is an atypical member of the ligand family of the FGF signaling system [1]. It acts as an endocrine factor with important roles in regulating the homeostasis of lipid, glucose and energy metabolism [2,3]. FGF21 directly elicits these effects through binding to a transmembrane protein complex consisting of a conventional FGF receptor (FGFR) tyrosine kinase and a co-factor betaKlotho (KLB) in adipocytes of the adipose tissue [4-10]. Hepatic FGF21 and adipose FGFR1-KLB constitute a negative regulatory axis for lipid, carbohydrate and energy metabolism in maintaining overall metabolic homeostasis. This is in parallel to the axis of ileal FGF19 to hepatic FGFR4-KLB for negative regulation of bile acid synthesis, and bone FGF23 to kidney FGFR-Klotho (KL) for negative regulation of mineral metabolism. In animal studies, overexpression or pharmacological administration of FGF21 ameliorates fatty liver, obesity and type 2 diabetes without a hyperproliferative side-effect characteristic of paracrine and autocrine-acting heparan sulfate-binding FGFs [3,11-13]. These effects are likely achieved by: (1) stimulating energy expenditure and futile cycling and regulating lipolysis, fatty acid oxidation and glucose utilization directly in white and brown adipose tissues; and (2) indirectly reducing lipogenesis and hepatosteatosis through enhancing triglyceride clearance, β-oxidation and ketogenesis in the liver [2,3,11-14].

Similar to FGF19 (FGF15 in mouse) and FGF23, the other two members of the FGF19 subfamily, these effects of FGF21 are determined by the tissue-specific expression and signaling of different isoforms of FGFRs and KLB. Recent studies indicate a specificity of FGF21 for FGFR1-KLB in adipose tissues [5,8,9,15]. On the other hand, the liver appears to be the primary source of circulating FGF21. In the normal fed state, the expression of FGF21 is only detectable at a low level in the liver. However, in response to fasting and starvation, a ketogenic diet, NAFLD, steatosis, obesity and type 2 diabetes, the expression of FGF21 is increased significantly [2,16-22]. Treatments with rosiglitazone, pioglitazone, metformin and other PPAR agonists induce hepatic FGF21 expression [19,23]. High-fat diets, which cause NAFLD and NASH, induce FGF21 in the liver [24-26]. Liver transplantation or HIV infection in liver increases serum FGF21 level [27,28]. Ablations of several functionally distinct proteins in hepatocytes, such as Foxo1, BDNF, gp78, Nrf2 and TBP-2 that result in metabolic and cellular abnormalities, coincide with increase of FGF21 expression [29-33]. At the intracellular level, ER stress, mitochondrial respiratory chain deficiency and an autophagy deficit also induce FGF21 expression [34-37]. Although some other studies suggest that FGF21 may be also expressed by extra-hepatic tissues under PPARγ or ATF2 control, such as white adipose tissue (WAT), brown adipose tissue (BAT), pancreas and skeletal muscle [38-40], the extrahepatic expression appears to be relatively low and occurs under specific stress conditions, and does not contribute to serum FGF21 levels as significantly and broadly as the liver.

The induction of hepatic FGF21 expression by diverse types of hepatic stress indicates that FGF21 is a stress-responsive hepatokine that is activated during liver pathogenesis and injury that impinge on its normal metabolic functions in behalf of the organism. Induced FGF21 in turn acts as a secretory signal that targets predominantly the adipose tissue adipocytes for assistances (compensation or reduction) in normalizing metabolic parameters in order to maintain lipid and energy metabolic homeostasis. This in turn serves to reduce the potentially damaging effects on the liver imparted by the stress. This idea is in concert with recent results indicating that, FGF21 of predominantly hepatic origin acts specifically on FGFR1-KLB in adipose tissue, as a primary endocrine axis for regulating both hepatic and systemic lipid, glucose and energy metabolism [5,6,8-10]. However, the breadth of the stress that remarkably activates hepatic expression of FGF21 is unclear. Induction of hepatic FGF21 expression may be a general property of functional hepatocytes in response to liver stress caused by not only metabolic extremes, but also tumorigenesis, liver damage and chronic diseases. To test the hypothesis that FGF21 is a hepatokine induced by general hepatic stress signals, here we investigate the induction of FGF21 in the liver under several major types of liver perturbation including liver injury and regeneration, chemical and genetic hepatocellular carcinogenesis (HCC) in both mouse models and human patient samples. Taken together with other studies, we conclude that FGF21 is an indicator of liver function and stress, and has the potential to be a predictive biomarker for liver function test, early diagnosis of liver cancer, other hepatic diseases and minimally-invasive clinical analysis. Increased serum FGF21 that originates from the liver under stressful conditions may serve in an inter-organ feedback communication network involving the liver and adipose tissue, which results in minimizing the damaging effects on the liver caused by the stress. In addition to several nuclear receptors, we implicate other stress response factors such as p53 and STAT3 in the regulation of hepatic FGF21 expression that warrant further investigation.

## Methods

### Mouse hepatocellular carcinoma models

Mice were handled in accordance with the principles and procedure in the Guide for the Care and Use of Laboratory Animals. All experimental procedures were approved by the Institutional Animal Care and User Committee (IACUC).

Diethynitrosamine (DEN)-induced HCC was done as previously described [41]. In brief, cohorts of male C57BL/6J mice two weeks after birth were injected with DEN intraperitoneally (IP) at 10 mg/Kg body weight. Control mice were injected IP with PBS. Liver tissues from the left lobe were collected at 0, 0.25, 2, 4, 6, 8, 10, 12 and 14 month after injection, minced and frozen at −80°C, or fixed overnight in 4% PFA in 1 × PBS. Paraffin-embedded tissue blocks were sectioned into 5 μm slides for H&E staining or immunohistochemical (IHC) analyses.

The LKB1^+/−^ and LKB1^+/−^p53^+/−^ mouse lines were prepared as described [42]. Liver tissues from wildtype and mutant mice at one year of age were collected for mRNA extraction and gene expression analyses.

Mouse strains with liver-specific ablation of the Hippo pathway component SAV1 (SAV^f/f^Alb^Cre^) or MST1 and MST2 (MST1/2^f/f^Alb^Cre^) by *Albumin* (*ALB*) promoter driven Cre were prepared as described [43]. Mouse liver tissues and sera from wildtype and liver-specific knockout mice at 6, 12 or 24 month were collected for gene expression and serum protein analyses.

Mice with liver-specific ablation of PTEN were generated by cross-breeding PTEN^lox/lox^ mice with ALB-Cre mice and liver tissues from PTEN^lox/lox^ and PTEN^lox/lox^Alb^Cre^ mice were collected at one year of age.

Mouse lines deficient in FGFR4 (FGFR4^−/−^) or KLB (KLB^−/−^) were prepared as described [8], and liver tissues were collected for gene expression analyses at one year of age.

### Partial Hepatectomy (PHx)

For 70% hepatectomy, C56BL/6J mice were anesthetized by inhalation of isoflurane. The left lateral and medium lobes were ligated and removed. Liver tissues (right lobes) and sera were collected at 0.5, 1, 2, 3, 4, 7 days post-surgery for mRNA expression and protein analyses.

### Gene expression analyses

Total RNA isolation, first-strand DNA synthesis, primer design and quantitative PCR analysis were performed as described [8]. Primer pairs for analyzing the expressions of *FGF21, FGFR4, KLB* and *ALB* genes are 5^′^-TTCAAATCCTGGGTGTCAAA and 5^′^-CAGCAGCAGTTCTCTGAAGC, 5^′^-CAGAGGCCTTTGGTATGGAT and 5^′^-AGGTCTGCCAAATCCTTGTC, 5^′^-CAGAGAAGGAGGAGGTGAGG and 5^′^-CAGCACCTGCCTTAAGTTGA, and 5^′^-ACCCCGAAGCTTGATGGTGTGAAG and 5^′^-GCAAGTCTGCAGTTTGCTGGAGAT respectively.

### Analyses of serum FGF21 protein levels

Serum was obtained from mice at the times as indicated in the text. Soluble KLB (sKLB) with transmembrane and intracellular domains replaced by 6 × His tag was produced in T-Rex 293 cells by tetracycline induction as described [44]. sKLB secreted into the culture medium was immobilized on Ni-Chelating beads. Aliquots of sKLB-bound beads were used to enrich FGF21 from mouse sera, which was then analyzed by western blotting with anti-FGF21 antibody (Cat #ab66564, Abcam Inc, MA) and quantified by densitometry.

Where indicated, plasma FGF21 concentration was also determined in duplicate by a mouse-specific ELISA kit (EMD Millipore, Billerica, MA) according to the manufacturer’s protocol in an effective range of 50–12000 pg/ml. The coefficient of variation was less than 10% within a same analysis and less than 8% between separate analyses.

### Immunohistochemical (IHC) analysis of FGF21 in mouse liver tissue section

Twelve mouse liver sections (5 μm) for each experimental condition were treated at 100°C to retrieve antigens in a pressure steamer containing 10 mM citrate buffer (pH 6.0) for 1 hr. The sections were de-paraffinized, hydrated and then immersed in 0.3% hydrogen peroxide for 20 min and incubated in 5 μg/ml anti-FGF21 antibody (Cat #ab66564, Abcam Inc, MA; http://www.abcam.com/FGF21-antibody-ab66564.html) containing 1 mg/ml BSA overnight. Second anti-rabbit IgG-Biotin and ExtrAvidin conjugated to peroxidase (Sigma-Aldrich, St. Louis, MO) were used for enzymatic colorigenic staining with AEC (N,N-dimethylformamide) as the chromogen. The section was then counterstained with haematoxylin and mounted with DPX. The slides were analyzed by a pathologist and photographed digitally by light microscopy.

### IHC analyses of FGF21 in tissue microarrays of normal and diseased human livers

Human liver tissue microarrays were obtained from US Biomax Inc, with an ethic statement, “All tissue is collected under the highest ethical standards with the donor being informed completely and with their consent. We make sure we follow standard medical care and protect the donors’ privacy. All human tissues are collected under HIPPA approved protocols. All samples have been tested negative for HIV and Hepatitis B or their counterparts in animals, and approved for commercial product development”.

The use of these commercially available and processed human tissue microarrays for research work followed Institutional Review Board (IRB) standards. IHC staining described above for expression of *FGF21* in human livers was performed on 5 μm unstained microarray slides (#LV1201 and LV803). The LV1201 array contains 25 HCC, 14 normal liver tissues, 16 fatty degeneration, 21 chronic active hepatitis, 30 cirrhosis, 3 cysts and 10 hemangioma cases. The LV803 microarray contains 26 sets of HCC with matched or unmatched tumor tissues and tumor adjacent phenotypically normal tissues.

### Luciferase reporter assay

Luciferase reporter constructs with human *FGF21* promoter sequence, FGF21 +5, -98, -997 and −1497 in TK-Luc were provided by Dr. Steven Kliewer (The University of Texas Southwest Medical Center) [2]. pFGF21 +11, -289, -443 and −1.6 K in PGL4.12 were from Dr. Yutaka Taketani (University of Tokushima, Japan) [45]. Wildtype and mutant constructs of p53 were from Dr. Weiqin Lu (University of Texas MD Anderson Cancer Center, University of Texas). Hep3B cells cultured in 12-well plate in DMEM high-glucose medium supplemented with 7% FBS, were transfected with these constructs with 15 μg/ml poly(ethylenimine) for 2 hrs. Empty vectors were used as controls. After further culture for 48 hr, the luciferase assay was performed according to manufacturer’s protocol using beetle Luciferin as the substrate in the presence of ATP and CoA (#E1500, Promega, Madison, WI).

### CHIP Assay

Chromatin immunoprecipitation (CHIP) analyses for p53 and STAT3 binding in the FGF21 promoter regions were done in Hep3B cells according to the manufacturer’s assay protocol (#17-371 EZ-CHIP, EMD Millipore). Lysates containing chromosomal DNA from about 1x10^6^ 95% confluent Hep3B cells were sheared to an average size of 1 to 5 kb fragments by ultrasonication and immunoprecipitated (IP) by anti-p53 (FL-393) (#SC-6243, Santa Cruz Biotechnology) and anti-pTyr705-STAT3 (#9131, Cell Signaling Technologies). Quantitative PCR using the SYBR Green JumpStart Taq Ready Mix (Sigma) on the Stratagene Mx3000P qPCR system was then applied to determine the binding sites of p53 and STAT3. Primers were designed to amplify about 200 bp encompassing the putative sites predicted by an *in silico* program based on the human FGF21 chromosome DNA sequence (http://www.sabiosciences.com/chipqpcrsearch.php). Normal mouse or rabbit IgG and anti-RNA polymerase II antibody were negative and positive controls, respectively. Lysates with sheared DNA without IP were used as sample input. Primer pairs 5^′^-AGACCCAGGAGTCTGGCC and 5^′^-GGGATAGATGCAGAAGCT, and 5^′^-CTCCAGAAGATGCCAGGC and 5^′^-CTCCAGAAGATGCCAGGC were used for analyses of p53 binding sites A (−191 to −215) and B (−6016 to −6026), respectively, by quantitative PCR. Primer pairs 5^′^-AGAGTTCCAGAGGAGGAT and 5^′^-AAGTGAGGCCCAGTGGGA, 5v-GCAGATAGTCCCGACGGC and 5^′^-GGAACAGATCCGCAGAGA, and 5^′^-GAGCCACGAAGTGGACAT and 5^′^-CCTCCGCGTGGGCAGAAG were used to identify STAT3 binding sites A (+2269 to +2276), B and C, respectively.

### Statistical analysis

Experiments were reproduced three times independently with triplicates in each experiment. Photomicrographs are representative of three or more experiments. Where indicated, the mean and standard deviation (sd) were reported. Comparisons between different genotype groups were performed with the unpaired *t* test. Values were deemed to be statistically significantly different at p ≤ 0.05.

## Results

### FGF21 is induced following mouse liver injury

**Figure 1:**
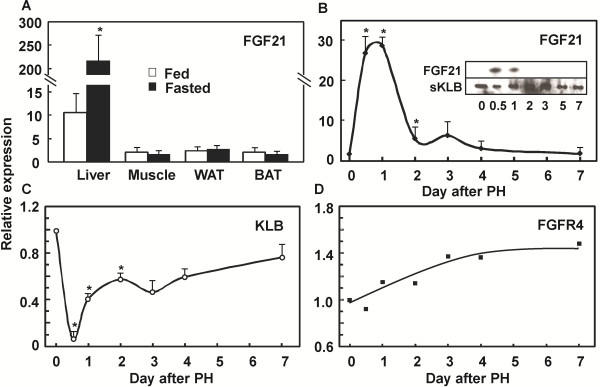
**Liver is the source of FGF21 elevation upon dietary restriction and liver damage.** The FGF21 expression level was analyzed by quantitative PCR in the indicated mouse tissues under different conditions. **A***:* Increase of FGF21 mRNA levels specifically in mouse livers after prolonged starvation for 48 hrs. mRNA levels are expressed as fold changes relative to the internal β-actin levels in the respective tissues. WAT: white adipose tissue. BAT: brown adipose tissue. Data are shown as mean ± sd, *p (n = 6) <0.05. **B***:* Induction of hepatic FGF21 mRNA in the liver after PHx. mRNA levels at different time lapses after PHx are expressed as fold changes relative to that before PHx. *p <0.05 (n = 3 for each data point). *Inset:* the corresponding serum FGF21 protein levels were determined by soluble KLB (sKLB) pulldown and western blotting analyses as described in the Method section. **C***:* Changes of hepatic KLB mRNA levels after PHx. **D***:* Changes of hepatic FGFR4 mRNA levels after PHx. Total RNA samples for analyses of KLB and FGFR4 expression are the same as for FGF21 in (**B**).

The expression of FGF21 is relatively low in the liver, and undetectable in muscle, WAT, BAT (Figure 1A), ileum and pancreas (not shown) in the fed state on a normal diet. After starvation for 48 hrs that induces hepatic steatosis, the expression of FGF21 was induced more than 220 fold exclusively in the liver (Figure 1A), which was about 22 times that in the fed state. Extrahepatic tissues did not exhibit such a remarkably inducible response to starvation.

To further test the idea that hepatic FGF21 expression indicates the functional status and perturbation of the liver, we first examined hepatic FGF21 mRNA after a partial hepatectomy that transiently reduces the full functional capacity of the liver until regeneration is complete. The expression of FGF21 was increased more than 30 times in the first 12 hrs to 2 day of regeneration after 1/3 PHx (Figure 1B), and gradually decreased in the course of liver regeneration and returned to normal basal levels after one week. The corresponding serum FGF21 was first enriched by binding to 6 × His tagged soluble KLB protein immobilized on Ni^2+^-Chelating beads [44], and then detected by antibody in western blot analysis. The levels of serum FGF21 protein (Figure 1B inset) followed the same pattern as hepatic mRNA levels determined by quantitative PCR analysis. In contrast, the expression of hepatic KLB followed an opposite pattern to that of FGF21 (Figure 1C). The expression of hepatic resident FGFR4 remained relatively unchanged (Figure 1D). The peak changes in the expressions of FGFR4 and KLB were about 1.4 and 0.09 times that of the livers before PHx, respectively. This indicates that FGF21 is an acute hepatic secretory factor in response to reversible loss of liver mass and functional capacity.

### FGF21 is induced in mouse hepatocytes during genetic hepatocarcinogenesis

**Figure 2:**
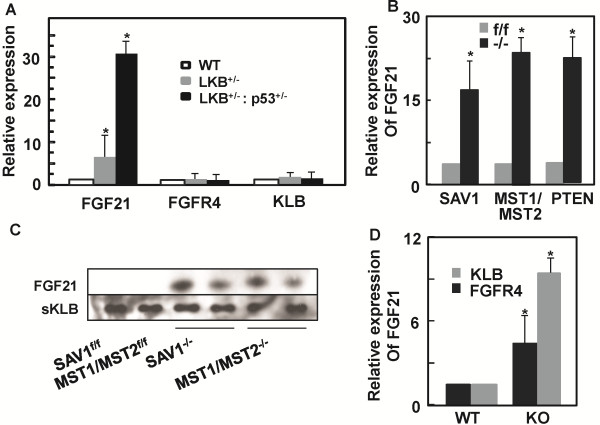
**Elevation of hepatic FGF21 expression during genetic hepatocarcinogenesis and perturbation.** Hepatic FGF21 mRNA levels were analyzed by quantitative PCR in the mouse liver tissues under different perturbation conditions at an age of one year. mRNA levels are expressed as fold changes relative to that in the wildtype or control mouse livers, which is assigned as an arbitrary unit 1. **A***:* Increase of FGF21 mRNA levels in mouse livers with haploinsufficiency for tumor suppressors LKB1 and p53. Changes in expression of FGFR4 and KLB were also measured in the corresponding tissues. *p < 0.05 (n = 6). WT: wildtype. **B***:* Increase of FGF21 mRNA levels in mouse livers with conditional ablation of tumor suppressors SAV1, compound MST1/MST2 or PTEN. f/f: floxed alleles for SAV1, MST1/MST2 or PTEN genes. −/−: ALB-Cre mediated hepatic deletion of SAV1, MST1/MST2 or PTEN. *p < 0.05 (n = 3-5 for each data point). **C***:* Changes in the corresponding serum FGF21 protein levels in mice used in (B) were determined as in Figure 1B *inset*. **D***:* Changes of hepatic FGF21 mRNA levels after whole-body knockout of KLB or FGFR4. *p < 0.05 (n = 6 for each data point). KO: whole-body knockout.

To determine whether FGF21 expression is induced in hepatocytes during hepatic tumorigenesis, we analyzed the expression of FGF21 in several HCC models. The tumor suppressor LKB1 heterozygous knockout mice developed spontaneous HCC upon aging [42,46]. The expression of FGF21 was increased about 6 fold in the LKB^+/−^ haploinsufficient livers (Figure 2A), and strikingly more than 30 fold in the compound p53^+/−^LKB1^+/−^ mouse livers compared to the wildtype counterparts. In contrast, the liver residents FGFR4 and KLB remain unchanged. Conditional hepatic ablation of Hippo pathway component SAV1 or compound MST1/2 also resulted in hepatic tumor formation. This was accompanied by an increase of 16 and 22 times in FGF21 expression in the livers of SAV^f/f^Alb^Cre^ and MST1/2^f/f^Alb^Cre^ mice, respectively (Figure 2B). The serum protein levels of FGF21 also followed the mRNA pattern (Figure 2C). Hepatic ablation of PTEN as a tumor suppressor and the PI3K/AKT signal controller, which resulted in fatty liver and hepatocellular carcinoma [47], also upregulated FGF21 expression at about 25 times in the PTEN^f/f^Alb^Cre^ livers than those of PTEN^f/f^ controls (Figure 2B).

Consistent with genetic alterations in genes that result in liver cancer, genetic deficiency that causes metabolic perturbations without tumorigenesis in the livers also induced hepatic FGF21 expression. Both FGFR4 and KLB are highly expressed in the liver and mediate the effects of FGF19 but not FGF21 [8,44,48]. Deficiency of either FGFR4 or KLB significantly disrupted the ability of liver to regulate bile acid homeostasis. We found that the expression of FGF21 was increased by 5 and 10 times in FGFR4^−/−^ and KLB^−/−^ livers, respectively, over the wildtype controls under these conditions (Figure 2D).

**Figure 3:**
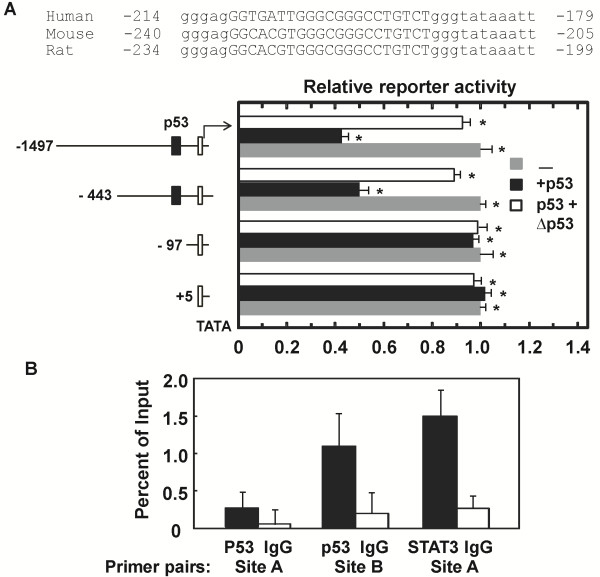
**Potential regulation of hepatic FGF21 expression by p53 and STAT3. A***:* Luciferase reporter activities with different FGF21 promoter sequences. Potential p53 binding locus and sequence conservation in the proximal region of FGF21 promoter were shown across human, mouse and rat species. Reporter assays were performed in Hep3B cells co-transfected with the indicated FGF21-luciferase reporter plasmids (−1497, -443 and −97) or control plasmid (+5) in the absence (gray bar) or presence of expression plasmids for p53 wildtype (black bars) and mutant (open bar). Cells were maintained in high glucose medium. Data are shown as mean ± sd, * p < 0.05 (n = 3 for each data point). **B***:* Determination of p53 and STAT3 binding regions. CHIP assay was performed as described in the Methods section in Hep3B cells. Antibody enrichment of DNA regions were analyzed by qPCR and expressed as percent recovery relative to the inputs. Normal IgG was used as negative control for p53 and active STAT3 antibodies. Values are means ± sd of duplicate PCRs performed at least three times with similar results, * p < 0.05.

Our data suggest that haploinsufficiency of p53 significantly affects FGF21 expression (Figure 2C). As p53 is a transcription factor and plays important roles in liver cancer, liver diseases and metabolic regulation, and loss of p53 function is known to contribute to tumorigenesis, it may regulate the expression of FGF21 gene under certain hepatic stress conditions, such as tumorigenesis. *In silico* analysis revealed an atypical p53 transcription factor binding site A, GGTGATTGGGCGGGCCTGTCT, at −191 to −215 bp upstream of the ATG translational start site of FGF21 gene (http://www.mybioinfo.info) (Figure 3A). This sequence in the proximal promoter region of FGF21 is conserved across human, mouse and rat species (Figure 3A). Luciferase reporter assay with the FGF21 promoter and upstream regions showed that p53 negatively regulate FGF21 expression in Hep3B cells cultured in high glucose medium (Figure 3A). This wildtype p53 effect was significantly abrogated by a mutant of p53, which is deficient in the N-terminal transcription activation domain and part of the DNA-binding domain (1-Met246 deletion) but still contains the C-terminal oligomerization domain (Figure 3A). These data support the idea that FGF21 is an independent indicator of genetic hepatocarcinogenesis. p53 may account for an additional or combinatory mechanism for regulating FGF21 expression in association with liver damage and carcinogenesis beyond metabolic alterations regulated by several nuclear receptors (NRs).

CHIP and quantitative PCR analyses revealed another potential p53 binding region surrounding putative site B of GAGACAAGTCT at −6016 to −6026 bp from the ATG site (Figure 3B) (http://www.sabiosciences.com/chipqpcrsearch.php). Among several predicted putative sites (see Methods section), the region surrounding site A of GGCTTCCC sequence at +2269 to +2276 bp was also found to bind STAT3 in these assays (Figure 3B). These results indicate that induction of FGF21 expression is potentially controlled by multiple transcription factors that presumably respond to different stress conditions.

### FGF21 is induced in mouse hepatocytes during chemical damage and hepatocarcinogenesis

**Figure 4:**
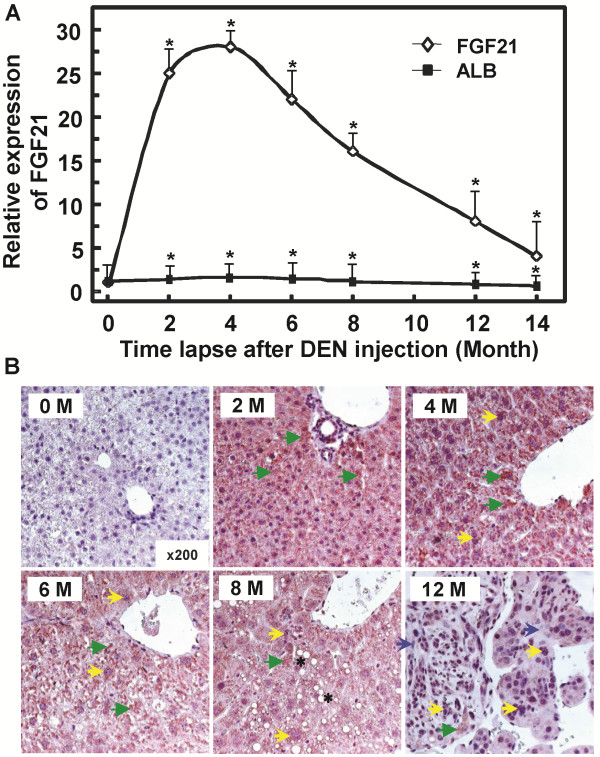
**Elevation in FGF21 expression during DEN-induced hepatocarcinogenesis. A***:* Temporal changes of hepatic FGF21 mRNA levels in mouse livers following initial treatment with DEN. FGF21 and ALB mRNA levels were analyzed by quantitative PCR in the mouse liver tissues during different time courses of DEN-induced tumorigenic effect. mRNA levels are expressed as fold changes relative to that injected with PBS, which is assigned as an arbitrary unit 1. *p < 0.05 (n = 3-4 for each time point). **B***:* Immunohistochemical analyses of FGF21 protein levels in mouse liver sections (cytosolic maroon stains) before DEN injection or 2, 4, 6, 8 and 12 month after DEN injection. There is only scattered staining in the sections from the livers at 12 months post-injection (green arrowheads). Data are representatives for FGF21 antigens from the livers of 3–5 mice at each time point. Green arrowhead: concentrated cytoplasmic FGF21 staining; Yellow arrow: enlarged or irregular nuclei; Black asterisk: lipid droplet; Blue arrow: fibrotic cirrhotic area.

Upon a single injection of hepatic carcinogen DEN at an age of two weeks to induce HCC, the expression of FGF21 increased significantly in the early stage when chemical damage is apparent before overt carcinogenesis is evident. The increases were apparent in the first week after treatment and have a peak induction of over 27 times at 2 to 4 months compared to mouse livers injected with PBS (Figure 4A). IHC analyses of FGF21 in mouse liver sections with anti-FGF21 antibody (#ab66564, Abcam Inc) following DEN treatment revealed a remarkable increase of FGF21 protein in the cytoplasm of phenotypically normal hepatocytes (green arrowheads) in the early and middle stages of hepatic carcinogenesis at 6–8 months, as compared to the same without DEN injection (0 month) (Figure 4B). The cytosolic maroon color staining was uniformly high in the sections from 2, 4, 6 and 8 months. There was no or only weak staining in the section before DEN injection (0 month), and only scattered weak stains in the sections of the livers treated with DEN for 12 months, where hyperplasia and tumor foci occurred. FGF21 expression was lost or attenuated in cells with abnormal and irregular expansion of nuclei (yellow arrows) accompanied by focal steatosis (black asterisks) and cirrhosis (blue arrows) in surrounding hepatocytes (Figure 4B. 6, 8 and 12 months). In contrast, the expression of hepatic albumin during the first six month period remained essentially unaltered, and exhibited only a 35% reduction at 14 months post DEN injection as compared to PBS control (Figure 4A). This indicates that FGF21 expression is lost as cells progress to malignancy, while the remaining scattered phenotypically normal hepatocytes adjacent to the hyperplasia or tumor foci still express FGF21 at highly elevated levels (Figure 4B. 12 month).

**Figure 5:**
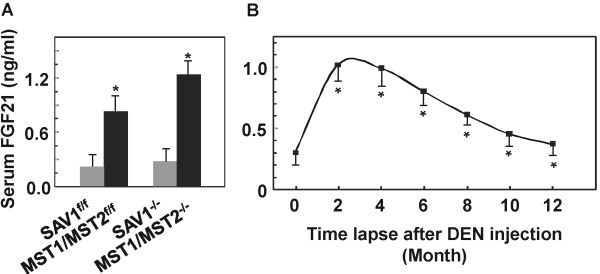
**Increases of serum FGF21 protein levels in mice undergoing hepatocarcinogenesis.** Protein levels in sera obtained from mice as indicated were determined by ELISA as described in the Methods section. 20 μl plasma from each mouse was analyzed in duplicate. **A***:* Changes of serum FGF21 levels in mice with deficiency in Hippo pathway components SAV1 and compound MST1/2 (Figure 2B). **B***:* Temporal changes of serum FGF21 levels in mice following DEN-induced hepatocarcinogenesis (Figure 4A). *p < 0.05 (n = 3-4 for each time point).

We further analyzed the serum levels of FGF21 as a hepatic hormone by ELISA upon hepatocarcinogenesis. A similar trend of increase was observed in the serum samples taken from the corresponding mice used for mRNA analyses (Figure 2B, 4A), albeit with much less extent of increase (Figure 5). Hepatic ablation of SAV1 and compound MST1/2 increased the serum FGF21 levels from 0.29±0.017 and 0.37±0.026 ng/ml in the control mice to 0.87±0.056 and 1.24±0.068 ng/ml in the mutant mice, respectively (Figure 5A). DEN treatment led to an increase of serum FGF21 protein levels similar to that of hepatic mRNA induction (Figure 5B, 4A). The peak serum levels were about 0.95-1.05 ng/ml at 2 to 4 months of age following DEN treatment, compared to 0.31 ng/ml in the untreated mice.

Our data indicate that FGF21 is a hepatokine induced in phenotypically normal hepatocytes upon acute carcinogen treatment and chronic carcinogenic transformation, and liver damage and partial resection resulting in reduced liver function. Changes in its hepatic expression or serum protein levels potentially mark the function of hepatocytes, but not malignant hepatocellular carcinoma cells that have lost their hepatocyte identity. These data concerning FGF21 expression are consistent with the notion that FGF21 is a property of differentiated hepatocytes and induced by agents perturbing normal liver that leads to liver tumorigenesis and disease pathogenesis. However, its expression is not a direct genetic marker of hepatoma cells per se.

### FGF21 expression is increased in regions surrounding the human hepatic lesions

**Figure 6:**
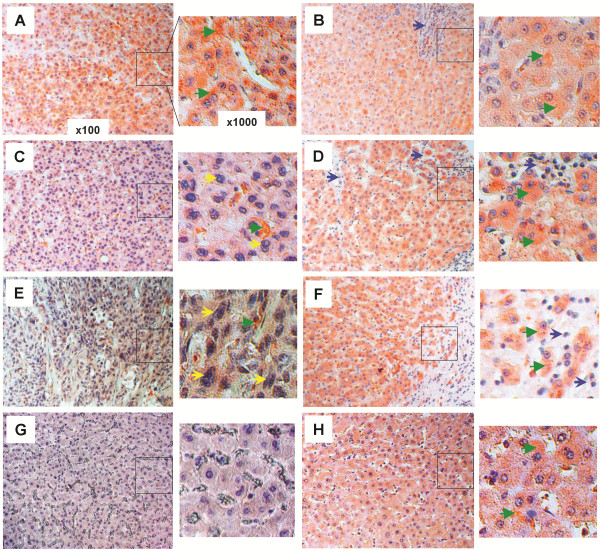
**Upregulation of FGF21 expression in human hepatic tissues during HCC development.** The expression levels of FGF21 were assessed by IHC (cytosolic maroon or orange stains) with anti-FGF21 antibody in the liver tissue sections from human patients as described in the Methods section. Representative images taken by light microscopy were shown for the following disease conditions: (**A**) grade 1 HCC tissues and (**B**) the tumor-adjacent (≥1.5 cm from the edge of tumor foci) hepatic tissues, (**C**) grade 2 HCC tissues and (**D**) the tumor-adjacent hepatic tissues, (**E**) grade 3 HCC tissues and (**F**) the tumor-adjacent hepatic tissues. This is in contrast to the normal hepatic tissues from healthy individuals (**G**), and phenotypically normal liver tissues (**H**) from the HCC patients. Low-magnification (×100) images are on the *left*, and black-boxed sections are enlarged (×1000) on the *right*. Green arrowhead: concentrated cytoplasmic FGF21 staining; Yellow arrow: enlarged or irregular nucleus; Blue arrow: fibrotic cirrhotic area.

**Figure 7:**
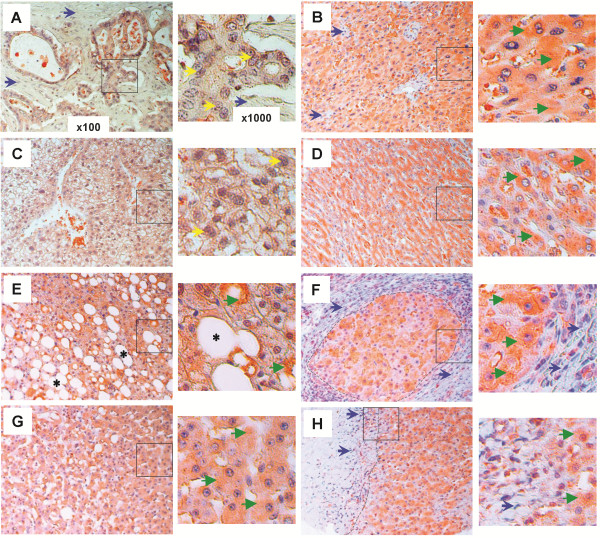
**Upregulation of FGF21 expression in human hepatic tissues undergoing other diverse pathologies.** The expression levels of FGF21 (cytosolic maroon or orange stains) were assessed by IHC as in Figures 4 and 6 in the liver tissues having the following disease conditions: cholangiocellular carcinoma (**A**) and the adjacent hepatic tissues (≥1.5 cm from the edge of tumor foci) (**B**), clear cell hepatocellular carcinoma (**C**) and the adjacent hepatic tissues (**D**), fatty degeneration (**E**), cirrhosis and the adjacent hepatic tissues (**F**), viral hepatitis (**G**), and liver cyst and the adjacent hepatic tissue (**H**). Low-magnification (×100) images are on the *left*, and black-boxed sections are enlarged (×1000) on the *right*. Green arrowhead: concentrated cytoplasmic FGF21 staining; Yellow arrow: enlarged or irregular nucleus; Blue arrow: fibrotic cirrhotic area; Black asterisk: lipid droplet; Black broken line: the boundary between large cirrhotic lesion and the surrounded hepatic tissue island (**F**), or between the fibrotic cyst and the hepatic tissue (**H**).

To evaluate the clinical significance of FGF21 expression in association with human HCC and liver diseases, we analyzed FGF21 expression by IHC analyses with anti-FGF21 antibody in clinically dissected, proven and graded liver tissue sections from human patients with known diverse causes of liver diseases. Among 46 human HCC and 5 cholangiocellular carcinoma sample sections examined, all grade 1 HCC areas of well-differentiated cells (Figure 6A) and all hepatocytes in tumor-adjacent (≥ 1.5 cm from the edge of the tumor foci) (Figure 6B, D, F) and phenotypically normal liver areas (as represented in Figure 6H from patients having different grades of HCC) from grade 1–3 HCC patients, exhibited a high level of staining for FGF21 (as represented by the green arrowheads). This was in marked contrast to the gradually diminished or lost expression of FGF21 in the HCC foci areas with grade 2 of moderately-differentiated (Figure 6C) and grade 3 of poorly differentiated tumor cells (Figure 6E). Normal hepatocytes (Figure 6G) in 10 out of 14 liver sections from healthy patients showed no or weak staining, and only 4 sections showed a moderately high level of staining with unknown causes. This was also evident in cholangiocellular carcinoma with bile duct epithelium proliferation (yellow arrows) (Figure 7A) and clear cell-type hepatocellular carcinoma (Figure 7C) with no or weak FGF21 expression in tumor cells; however, FGF21 was highly expressed in fields adjacent to tumors (Figure 7B, D) and in phenotypically normal hepatocytes (as represented in Figure 6H). Cirrhosis was often observed in otherwise phenotypically normal areas adjacent to tumors (Figure 6B, D, F; Figure 7B, D).

Hepatocytes in livers showing fatty degeneration (black asterisks) from all 16 patients (Figure 7E), and hepatocytes or regenerative hepatic tissues (shown inside the black broken line) containing nodular cirrhosis or fibrotic lesions (blue arrows and outside the black broken line) from all 30 patients (Figure 7F), exhibited an intense FGF21 signal. The cells within cirrhotic and fibrotic foci showed no expression of FGF21. Nineteen of 21 liver sections with chronic or active viral hepatitis inflammation showed a comparably high level of FGF21 signal to that of Figure 7G, and the rest showed a weaker but still stronger signal than livers from normal healthy individuals. FGF21 expression was also high in hepatic tissues (right of the black broken line) adjacent to the wall of liver cysts, but not the cells in cyst lesions (left of the black broken line) (Figure 7H). Taken together, these data show that the mouse models recapitulated the findings in human liver diseases, and FGF21 expression is significantly induced in hepatocytes in response to perturbation of liver functional capacity by liver damage (viral infection, cirrhosis, steatosis and toxins), partial resection and carcinogenic transformation (HCC, clear cell HCC and cholangiocellular carcinoma).

## Discussion

In this survey, we analyzed the nature of the stress that activates the expression of hepatic FGF21. We found that the expression of FGF21 was significantly induced in the liver following reversible perturbation such as partial hepatectomy and regeneration, hepatosteatosis as well as irreversible hepatic damage from chronic hepatitis, cirrhosis, and chemical and genetic hepatocarcinogenesis in mouse models and human patient samples. Our data suggest that FGF21 is an inducible stress-sensing hepatokine, and its expression is associated with the loss of normal functional capacity of hepatocytes due to pathogenic processes. Our data are also supported collectively by other spontaneous reports indicating that FGF21 is preferentially induced in the liver upon fasting and starvation, steatosis, obesity, type 2 diabetes and genetic deficiency of specific genes in hepatocytes [2,16-21,23-27,49]. Taken together, we conclude that FGF21 is a novel hepatokine and marker for the functional status of mature/differentiated or phenotypically normal hepatocytes during the process of liver injury, recovery and pathogenesis. Since serum FGF21 originates predominantly from the liver in all these conditions, it is potentially a minimally-invasive biomarker for diagnosis and follow-up of clinical conditions including hepatocarcinogenesis, fatty degeneration, chronic and hepatitis inflammation, and liver damage and regeneration in general. Under carefully controlled test conditions, hepatic or serum levels of FGF21 could be an independent biomarker of liver pathological changes, or a combinatory biomarker with other existing biomarkers, such as the aspartate aminotransferase/alanine aminotransferase ratio (AST/ALT). Our results provide strong rationale for extensive clinical validation of the association and standardization of test conditions.

Several reports have shown that hepatic expression of FGF21 is regulated by nuclear receptor PPARα/RXRα upon fasting and starvation that change the levels of fatty acids, the natural ligand of PPARα, or by treatment with PPARα agonists [2,16,50]. This is similar to other NRs that regulate the expression of the other two members of the endocrine FGF subfamily under diverse conditions [51]. FGF19 induced by postprandial bile acids and ligands of the farnesoid X receptor (FXR) in the ileum regulates distal hepatic cholesterol/bile acid synthesis [51] and systemic lipid metabolism [52]. Vitamin D and the vitamin D receptor regulate FGF23 expression in bone, which in turn negatively impacts mineral metabolism in the kidney [53]. Other studies indicate that hepatic FGF21 expression is also regulated, either positively or negatively, by ChREBP, PPARγ, LXR or FXR/RXRα in diverse conditions [17,45,54,55]. In this study, we show that beyond NRs, the stress regulators p53 and STAT3 may also participate in the regulation of FGF21 expression. Deletions, mutations or change in the expression of p53 or STAT3 in hepatocytes contribute to hepatocellular carcinoma (HCC) or liver damage [56,57]. This is consistent with our finding that FGF21 expression is regulated by wildtype p53 and STAT3. Therefore, our results may indicate the existence of multiple mechanisms for regulating the expression of FGF21 in association with the differentiated function of hepatocytes, and damage or loss of liver function under diverse stress conditions. This new roles of p53 and STAT3 are in harmony with the many roles of p53 and STAT3 in cellular stress responses that impact cell growth, survival, death and metabolic homeostasis. As metabolic alteration and cell growth control are intertwined, p53 or STAT3 control of FGF21 expression could be cooperatively engaged with PPARs or other NRs that are master response and regulatory factors for metabolic abnormalities.

As liver is the central organ for maintenance of metabolic homeostasis, we expected that these stress conditions, which alter metabolic functions of the liver either directly through glucose/fat accumulation or indirectly through cellular abnormality including tumorigenesis, injury and damage, ultimately alter hepatic metabolites. These metabolites, such as FFA, cholesterol or even TG, are the output of normal liver function, and are proven pivotal activators or inhibitors of NRs that in turn regulate FGF21 expression. This is likely one of the major or direct mechanisms through which hepatic FGF21 expression is regulated. This idea is supported by observations that the development of many metabolic and cellular liver diseases from diverse causes undergoes a stage of lipid accumulation or steatosis. NRs in conjunction with other regulators some of which we reveal in the current study may be the unifying mechanism by which hepatic FGF21 expression in PHx, DEN treatment, hepatocarcinogenesis and other hepatic stress conditions is regulated. It is well-established that both p53 and STAT3 can collaborate with NRs under many of these stressful conditions.

Our analyses of liver tissue sections from human patients with diverse liver diseases further confirmed the notion that FGF21 is a potential biomarker of human liver diseases. Although it may not directly contribute to the disease etiology, its expression level reflects the function of phenotypically normal and mature hepatocytes and the functional status of the liver as a whole. The effect of its elevation is to counteract the overload and potentially damaging effect in both liver and the entire organism imposed by the stress. Our previous studies suggest that this is through a mechanism by which hepatic FGF21 targets extra-hepatic adipocytes and adipose tissues via an endocrine mechanism for compensatory metabolic regulation and alleviation of metabolic diseases including fatty liver, obesity and diabetes [5,6,8-10]. An increase in hepatic FGF21 mRNA expression correlates closely with the serum protein level (Figures 1, 2, and 5). This indicates that serum FGF21 may be an excellent minimally-invasive biomarker that is sensitive, specific and of predicative value for test of liver function and diagnosis of the onset, stage and prognosis of various liver diseases. The next step should be the study in a clinical setting.

**Figure 8:**
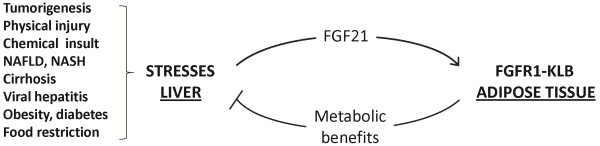
**Plausible action and role of hepatic FGF21 induced by multiple types of stresses.** Hepatic cellular and metabolic stresses imposed by injury and pathologies in the liver induce elevated expression of FGF21. FGF21 released from hepatocytes activates FGFR1-KLB complex in the peripheral adipose tissues. This results in metabolic benefits including correction of abnormal metabolic parameters and metabolic insufficiency resulted from perturbation of normal liver functions. As a result of this feedback response, potential damages imparted by the stress to the liver are attenuated or prevented.

Until recently, diverse extra-hepatic tissues were suggested as direct targets of FGF21 via an FGFR. Although most reports focused on effects of systemic FGF21 on glucose uptake [3] and lipolysis in fat tissue and isolated adipocytes [14,58,59], some suggested that FGF21 may directly regulate, through FGF21 signaling in hepatocytes, the responses of the liver to fasting and ketogenic diet, hepatic insulin sensitivity, triglyceride clearance and hepatosteatosis [2,11,16,60]. Others argued that effects of systemic FGF21 on liver were indirect [6,14,61,62]. This notion has been recently confirmed by direct genetic manipulation of FGFR isotypes in adipocytes and hepatocytes. Ablation of KLB or an adipose tissue-specific deficiency of FGFR1 and FGFR2 indicates that adipose tissue and more specifically adipocyte FGFR1, but not the liver, is the direct and predominant target of serum FGF21 action [8]. Our studies in these mouse models with DIO and administration of FGF21 further precipitated a consensus that the entirety of metabolic actions and pharmacotherapy effects of FGF21 is predominantly, if not solely, governed by the adipose tissue (both brown and white adipose tissues) FGFR1-KLB [10]. In all cases, elevation of FGF21 or treatment by FGF21 ameliorated hepatic steatosis and other abnormal hepatic metabolic parameters. Therefore, secreted hepatokine FGF21 appears to serve a beneficial function to the organism systemically and to the liver locally when the liver is under stress and cannot fully perform its normal function in metabolic homeostasis (Figure 8). This is through the axis from hepatic FGF21 to adipose FGFR1-KLB, and likely a secondary axis from adipose to the liver through metabolites and adipokines (such as adiponectin) [4,8-10] (Figure 8). Such an endocrine regulatory axis initiated by stress-responsive FGF21 results in concurrent attenuation of adipose lipolysis, hepatic lipogenesis and hepatosteatosis, and ultimately of stress-imposed liver damage [9,10].

Interestingly, at the intracellular level, FGF21 can be also significantly upregulated upon mitochondrial dysfunction and ER stress (Lu W et al., unpublished data). This is consistent with emerging studies in muscle with mitochondrial dysfunction and genetic disease indicating a possibility that FGF21 could be induced in muscle under metabolic and energy stress [34,36,37], which also targets the adipose tissue. This further supports a general role of FGF21 as a stress-responsive metabolic regulator that modulates cellular energy homeostasis in crisis. It would be interesting to know whether there is coordination between the liver and muscle for inducing FGF21 expression and contributing to serum FGF21 levels under stress. In respect to the hepatic biomarker utility, it is therefore important to define a test condition under which the muscle has a minimal or no input for FGF21 expression. The importance of non-hepatic FGF21 in muscle or other tissues under stress conditions and its coordination among adipocytes, the primary target for FGF21, and liver should be an area of fruitful future study.

Hepatic expression and blood levels of FGF21 under normal fasting and feeding cycles or a 48 hr fasting are low and vary widely among individuals in man. This characteristic of human FGF21 differs from rodent FGF21 that can be induced significantly by a 48 hr fasting or ketogenic diet [50,63]. The inducible expression of FGF21 in muscle under muscular stress in addition to the liver may also contribute to the wide interindividual variation of fasting levels of human serum FGF21. However, human serum FGF21 can be consistently elevated during extreme fasting (7 days) or starvation. Furthermore, both human and rodent FGF21 appear to be inducible to a high and relatively stable level under multiple stress conditions. All these facts further highlight the role of FGF21 as a stress-responsive factor.

Lastly, we show that both during transient regeneration in response to injury and in hepatomas, the expression pattern of the metabolic co-factor KLB is opposite to that of FGF21 in liver. This opposite pattern of FGF21 and KLB expression with no consistent change in FGFR4 may indicate a cellular KLB-free state in the early regeneration phase of the damaged liver, and is consistent with our contention that in normal physiology the FGFR4-KLB partnership is a negative regulator of hepatocyte proliferation as well as progression to hepatoma [41,44]. Depression of KLB may be essential to relieve the restriction on hepatocyte expansion during normal response to injury and restoration of normal liver physiology imposed by the FGFR4-KLB partnership, of which the primary function is to regulate bile acid synthesis.

## Conclusion

Taken together, our data support the idea that FGF21 is a stress-activated hepatokine and induced significantly in the liver upon perturbation and disease development. This stress-activated FGF21 expression may underlie the generally beneficial effects of FGF21 through alleviating liver overload such as steatosis and counteracting potential liver damage imposed by a variety of metabolic and cellular stresses [9,10] (Figure 8). Serum FGF21 levels likely reflects the original liver production source and is expected to be a biomarker for functional status of the liver and liver damage leading to hepatoma and disease of liver dysfunction. Liver biopsy has been the gold standard yet imperfect and invasive method with risks. There are also serious limitations for the existing invasive biomarkers, such as those used in FibroTest-ActiTest, AST/ALT and AST/platelets ratio index (APRI). These limitations may include poor sensitivity and specificity, indeterminate ranges and poor predicative values [64]. New biomarkers with better predictive values are needed. Future clinical experiments are needed to directly compare the clinical utility of serum FGF21 or combinatory utility of FGF21 with these existing biomarkers.

## Competing interests

Authors of this study declare no any potential conflict of financial interest or otherwise.

## Authors’ contributions

CY, WLM and YL designed research. CY, WL, TL, PY, MY, YH, XJ, CW and YL performed research. WL, FW, MHL, SCJY, RLJ, CW, RYT, MLF contributed reagents and reading of manuscript. CY, YL and WLM analyzed data. CY, WLM and YL wrote the paper. All authors read and approved the final manuscript.

## Pre-publication history

The pre-publication history for this paper can be accessed here:

http://www.biomedcentral.com/1471-230X/13/67/prepub
